# Ion Chromatography with Pulsed Amperometric Detection for Determining Cyanide in Urine and Meconium Samples

**DOI:** 10.3390/molecules26154672

**Published:** 2021-08-02

**Authors:** Ewa Jaszczak-Wilke, Krystyna Kozioł, Bogumiła Kiełbratowska, Żaneta Polkowska

**Affiliations:** 1 Department of Analytical Chemistry, Faculty of Chemistry, Gdansk University of Technology, 11/12 Narutowicza Str., 80-233 Gdansk, Poland; ewajaszc@student.pg.edu.pl (E.J.-W.); krystyna.koziol@pg.edu.pl (K.K.); 2 Department of Obstetrics, Medical University of Gdansk, Marii Skłodowskiej-Curie 3a, 80-210 Gdansk, Poland; kielbrat@wp.pl

**Keywords:** urine, meconium, tobacco smoking, cyanide, ion chromatography, PLE

## Abstract

The parents’ addictions and eating habits have a significant influence on the child’s growth. The first stool of a newborn baby provides a large amount of information about xenobiotics transmitted by the mother’s body. The analytical technique used in the study is ion chromatography with pulsed amperometric detection (IC-PAD). The biological samples, which were obtained from women staying in a maternity ward and their partners, revealed cyanide concentrations in urine samples spanning 1.30–25.3 μg L^−1^. Meanwhile, the results of the meconium samples were in the range of 1.54 μg L^−1^ to 24.9 μg L^−1^. Under the optimized chromatographic conditions, the IC-PAD system exhibited satisfactory repeatability (R < 3%, *n* = 3) and good linearity in the range of 1–100 μg L^−1^. Thus, it proved to be an effective tool for monitoring trace cyanide concentration in a series of human body fluid matrices, including meconium. Based on the literature review, this is the first application of the IC-PAD analytical technique for the determination of cyanide ions in meconium samples.

## 1. Introduction

Cyanide pollution is associated with human activity such as mining and developing the galvanic and chemical industries [1,2,3]. Humans also introduce cyanides into their bodies as a result of smoking tobacco products, exposure to environmental tobacco smoke (ETS), or living on a diet rich in cyanogenic glycosides [4]. Amygdalin is one of the cyanogenic glycosides widely available in edible plants such as almonds, apple seeds, and cucumbers. Upon their ripening or crushing, amygdalin is hydrolyzed to benzaldehyde and hydrogen cyanide [5,6,7]. Excessive consumption of these seeds may have a negative effect on the body and cause a number of adverse reactions such as diarrhea, vomiting, abdominal pain, and, in extreme cases, it may lead to death. The basic activity of cyanide consists in combining with trivalent cytochrome oxidase iron, which is the key enzyme of the respiratory chain [8]. The consequences of smoking are well known; they include various adverse health effects not only for smokers but also for those exposed to ETS. Exposure of pregnant women to the substances contained in ETS may lead to neurodevelopmental disorders in children. These are considered to be the result of changes in the infant’s brain brought about by fetal hypoxia. The compound primarily responsible for this is carbon monoxide in tobacco smoke. Another effect of active smoking whilst pregnant, or even passive exposure to toxic substances in the smoke, is the reduced birth weight of the fetus. This effect is marginal in the first trimester of pregnancy and increases as the pregnancy progresses, becoming the most significant in the third trimester. In addition to the reduced weight, other consequences evident in the postnatal phase include hyperactivity, reduced concentration, poor response to auditory stimuli in children in the first week of life, and lower intelligence in preschool age [9]. Despite this, a large number of women remain unable to quit smoking during pregnancy or are exposed to ETS from their partner or their environments.

Human biological material is an excellent source of information on human exposure to the variety of chemical substances present in the environment. The most common types of biological material used in analytical work are blood and urine because of the relatively easy way in which they may be collected. Additionally, taking urine samples does not require any interference in the human body, and they provide comprehensive information on the organism’s exposure to xenobiotics in recent days [10]. Collecting meconium samples is also non-invasive, and this material provides information on the exposure of the newborn throughout pregnancy [11].

Several methods have been published for the identification of cyanide or thiocyanide (metabolites) in different biological matrices including urine [12], breast milk [13], blood [14], saliva [15], and meconium [16]. Most of the recent literature describes determination of cyanide in whole blood samples collected during an autopsy in cases of suspected intoxication with cyanide or suicide [17,18]. Due to its ability to provide information over the course of the entire pregnancy, meconium has been receiving increasing attention. The most common methods for extracting analytes from a meconium sample are liquid–liquid extraction (LLE) and solid-phase extraction (SPE) [11].

In this work, we propose a simple and fast method for meconium sample preparation: pressurized solvent extraction (PLE). The PLE technique is useful for the isolation of compounds at trace level, which is convenient for analyzing meconium samples. Moreover, water was introduced as the extraction medium, which is in accordance with the principle of green chemistry as it limits the use of organic solvents [19]. Depending on the analytes determined, GC-EI-MS (for fatty acid ethyl esters (FAEEs)) [20], LC-ESI-MS/MS (for drugs of abuse and their metabolites) [21], LC-APCI-MS/MS (for pharmaceuticals) [22], GC-EI-MS (for volatile organic compounds (VOCs)) [23], and ICP-MS (for metals) [24] were used for the analysis of meconium. In this work, we propose a simple and efficient method for determining the cyanide content in biological samples: ion chromatography with pulsed amperometric detection (IC-PAD). The application of ion chromatography in the analysis of biological samples is characterized by high sensitivity and low detection limits [13].

To our knowledge, only very limited data have been published on meconium analysis. The purpose of this study was to answer the question of whether the baby’s meconium would reflect the tobacco addiction and eating habits of the parents. While the placenta provides a preview of the course of the pregnancy, it is uncertain whether such information can be obtained from analysis of a meconium sample. The aim of the study was to use meconium samples collected from newborn babies and urine samples collected from their parents to assess fetal exposure to cyanide ion. Samples of biological materials were collected from parents who may have been exposed to environmental tobacco smoke. The PLE extraction method developed here is sensitive, inexpensive, and environmentally friendly. After the appropriate sample preparation, the cyanide levels were determined by ion chromatography coupled with pulsed amperometric detection.

## 2. Materials and Methods

### 2.1. Study Objectives

The research group consisted of twenty-two pairs of parents (mothers and fathers) and their newborn children, with the exception that four of these pairs were tested before the birth. The donors anonymously completed a questionnaire concerning their diets and tobacco addictions (Table 1).

In brief, out of twenty-two donors, four of them were tested before giving birth. Most of the donor-mothers were 21–30 years old and received higher education. Five of them were active smokers and when the pregnancy was confirmed only one did not quit smoking. Three volunteers were passively exposed to smoke at home. Half of them were on thyroid medication. Much like the fathers, the mothers’ diets were rich in dairy products and almonds. Most of the donor-fathers were 31–40 years old and received higher education. Six of them declared active smoking up to the period of 10 years. However, only one of them admitted to smoking in the presence of his pregnant partner. None of the parents were exposed to fire smoke. Of the twenty-two infants, eighteen were born after 38 weeks of pregnancy and twenty of them received the Agpar score of ten (only two infants were assigned eight and nine points). All the newborns excreted meconium in the first day of life, within the first 3 h.

### 2.2. Sample Collection and Preparation

A total of 35 samples were collected between May and August 2018 from healthy volunteers. The urine samples were provided by parents of newborn babies and the meconium samples were obtained within 3 days of birth and stored at −20 °C until the analysis was performed. The donors completed an anonymous questionnaire in which there were questions relating to diet and tobacco addiction during the course of the pregnancy. Based on the questionnaire output, the donors were divided into three groups: active smokers (women who actively smoke tobacco during pregnancy), passive smokers (pregnant women who were exposed to tobacco smoke at home or at work), and the control group (where none of the parents were active or passive smokers).

**Figure 1:**
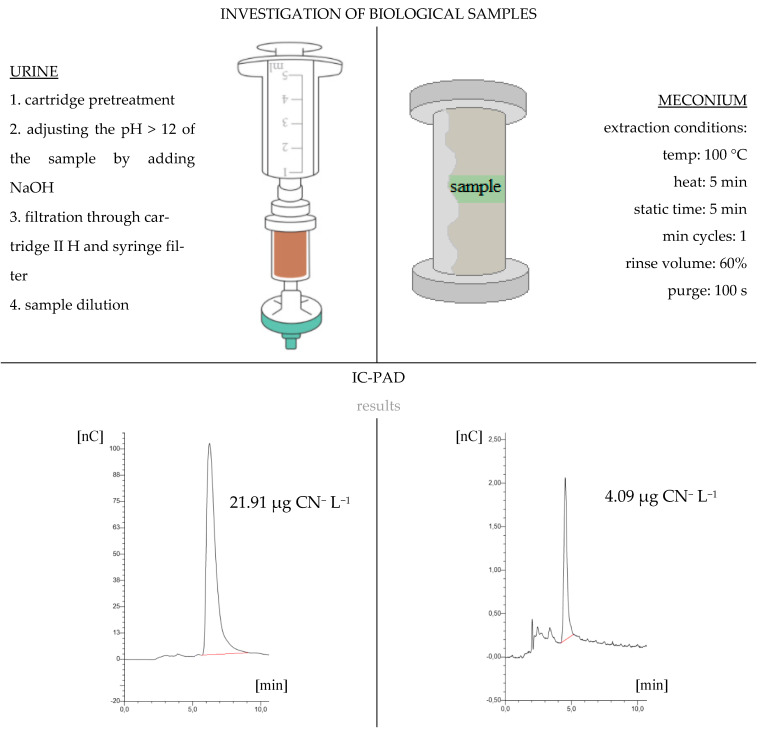
Diagram of the analytical procedure for determining CN- by using IC-PAD.

The urine samples were analyzed using the previously reported method [13]. The cyanide ion was extracted from the meconium samples using PLE at a temperature of 100 °C for 5 min of static time. The extraction column was filled with diatomaceous earth. Deionized water was used as the extraction medium in order to reduce the use of organic solvents in accordance with the principles of green chemistry. A 0.1 g meconium sample was extracted (Figure 1). The supernatant was collected and loaded into a preconditioned column (Cartridge II H, ThermoScientific, Waltham, MA, USA, 1-cc) to remove the interfering ions that can cause ion suppression during the analysis. The extract was filtered through a 0.2 µm nylon filter prior to IC-PAD analysis. All the samples were analyzed, at a minimum, in triplicate. The sodium cyanide (95% analytical grade) and sodium hydroxide (50% analytical grade) used in this work were supplied by Sigma Aldrich (St. Louis, MO, USA). All the dilutions and blanks were prepared with ultrapure deionized water produced by Mili-Q Gradient A10 System (18.2 MΩ, 25 °C).

### 2.3. IC-PAD Parameters

The cyanide ion analysis was performed on a Dionex ICS 3000 ion chromatograph (Dionex, Sunnyvale, CA, USA) equipped with a gradient pump, an autosampler, a column oven, a degasser, and a pulsed amperometric detector (consisting of an auxiliary electrode, i.e., a miniature flow cell with a titanium body, an Ag working electrode, and an Ag/AgCl reference electrode). The PAD recommended waveform for cyanide was as follows: (0 s, −0.1 V), (0.2 s, −1 V), (0.9 s, −0.1 V), (0.91 s, −1 V), (0.93 s, −0.3 V), (1 s, −0.3 V), the setting which is also suitable for the detection of thiosulfate, sulfide, and bromide. The separation was performed by a Dionex IonPac AS15 anion-exchange analytical column (ThermoScientific, 2 × 250 mm, 7.5 μm particle size) and protected with a Dionex IonPac AG15 anion-exchange guard column (ThermoScientific, 2 × 50 mm, 7.5 μm particle size). Both columns were equilibrated with 63 mM sodium hydroxide eluent. The eluent flow-rate was set at 0.25 mL min^−1^ and the injection volume equaled 40 μL. All measurements were taken at 30 °C with a column backpressure of approximately 1100 psi (7.58 MPa). For the cyanide content analysis, the conditions allowing for the best resolution of the separation were selected. Prior to the analytical runs, a calibration curve was prepared from an NaCN standard solution. The compound identification was based on the comparison of retention time with the standard solution runs. The analytical data were acquired and analyzed using Chromeleon software (version 6.8).

The procedure for determining cyanide ions in biological samples was validated to ensure the appropriate level of quality control and quality assurance of measurements [25]. Validation parameters for the determination of cyanide ions in urine samples were checked and were at the same level as the previous validation (Table 2). The linear range of the method was 1–50 µg L^−^^1^ of an NaCN standard solution with an R value of 0.986. The limit of detection for the cyanide ion is 0.58 µg L^−^^1^. Recovery was measured after the addition of two different concentrations (5 µg L^−^^1^ and 20 µg L^−^^1^) of an NaCN standard into the meconium samples. The average recoveries ranged from 79% to 83%. The good repeatability of the method is indicated by the CV, which was less than 3%. Possible interferences such as sulfides and nitrates were eliminated by using dedicated SPE columns [26].

## 3. Result and Discussion

**Figure 2:**
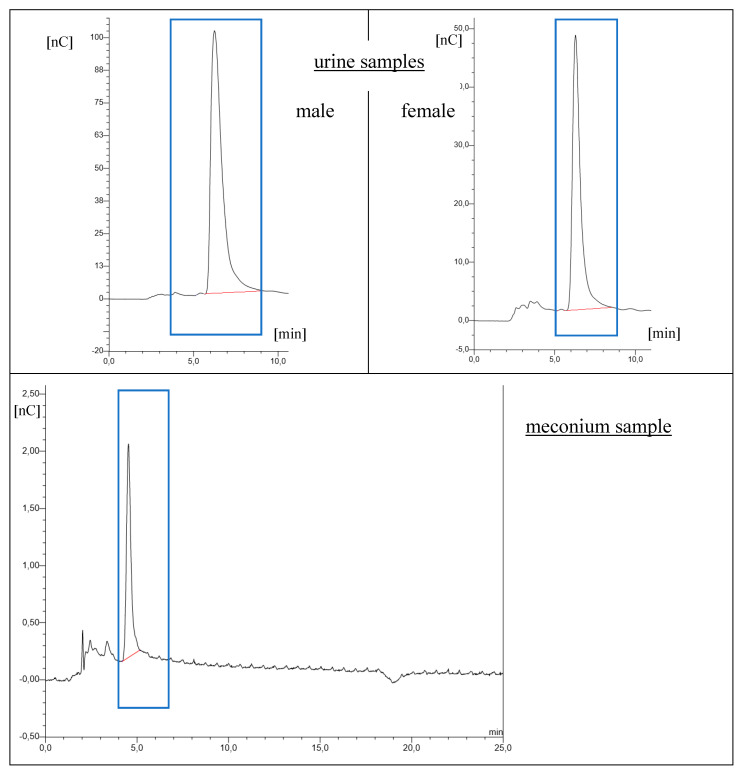
Example chromatograms of a tested smoker’s urine (father 21.91 µg L^−^^1^, mother 8.69 μg CN^−^ L^−1^), and a meconium sample (control group 4.09 µg L^−^^1^).

The IC-PAD method was successfully applied to determine the amount of cyanide ion in the urine and meconium samples (Figure 2). This technique is based on the measurement of the current as a function of time by applying a constant potential to the working electrode. On the surface of the working electrode the redox reactions of the analytes take place by means of a potentiometer, where the output current is proportional to the concentration of the analyte. The detector is designed as a thin layer system, where eluent flows in a channel parallel to the flat surface of the reference electrode. The resulting flow minimizes the noise. The design of the detector minimizes the electrical resistance between the working electrode and the auxiliary electrode by placing the auxiliary electrode across the narrow working electrode channel. The working electrode’s current is processed using low-noise amplifiers and analogue filters. The reference electrode is a standard combination electrode containing a glass membrane shelf and an Ag/AgCl shelf. The combined pH electrode monitors the reaction of the eluent. With an eluent pH of 7, the reference potential of the whole electrode is the same as that of the Ag/AgCl half-cylinder. When the pH value of the eluent increases, the potential of the shelf is reduced by 0.059 V per pH unit, e.g., when pH = 12, the potential is −0.295 V [27].

The cyanide ion is typically extracted from biological samples using polar extraction solvents. The solid-phase extraction method gives good results in the extraction of cyanide ion from urine samples. However, for meconium samples it was necessary to use PLE. The extraction column was filled with diatomaceous earth to protect the sample from overly high pressure.

Cyanide concentrations were determined in 11 and 13 urine samples of the mothers and fathers, respectively. Cyanide concentrations in meconium were detected in only eight samples (Table 3). Regarding the recently published studies [13], cyanide concentrations in mothers’ urine samples are lower. However, previous studies were conducted on a much larger number of actively smoking mothers (even during pregnancy).

### Correlations between the Levels of Cyanide in Parents’ and Newborn’s Biological Samples

**Figure 3:**
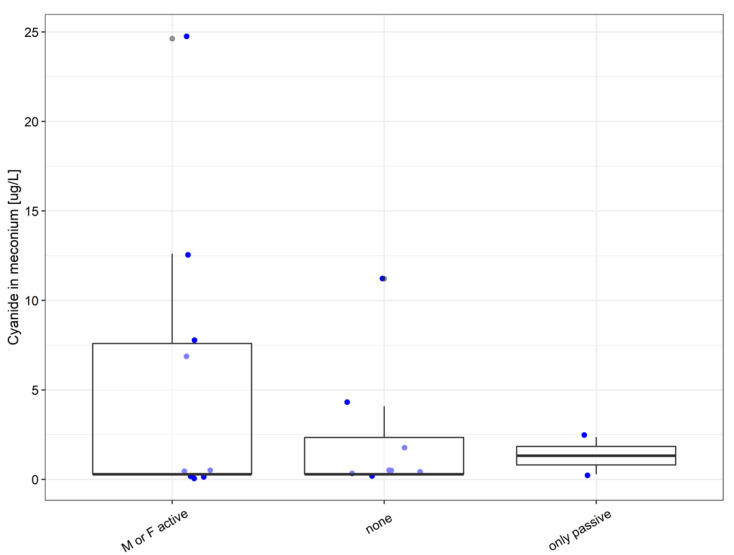
Smoking habits of the father or mother (F or M)—influence on the child’s meconium cyanide concentration. Differences between groups were not significant (Kruskal–Wallis ANOVA *p* = 0.76).

Statistically, no significant difference appeared between the concentrations of cyanide in the meconium sampled from the children of active smokers, passive smokers, and non-smokers (Figure 3).

Even if the data obtained from both fathers and mothers were pooled to increase the power of the statistical analysis (meaning the number of cases increases from 19 to 38), neither smoking status nor medication have a significant effect on cyanide concentrations in the urine of their newborn children. Hence, these two factors are unlikely to exert influence over the child’s organism. This may be due to a detoxification reaction that produces thiocyanide ions, whose toxicity is 200 times smaller than that of cyanide ions as they are easily soluble in water and excreted from the body along with bodily fluids.

**Figure 4:**
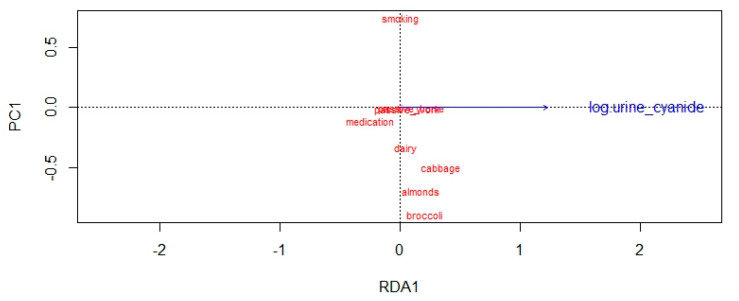
Redundancy analysis to discover important qualitative factors driving the cyanide concentrations in parents’ urine.

Another interesting variant which potentially affects the results is the diet. There was a slight tendency noted for higher median concentrations of cyanide in the urine of parents whose diet was rich in broccoli, cabbage, or almonds, yet none of these tendencies produced a significant inter-group effect which would correspond to the Kruskal–Wallis ANOVA *p*-value < 0.05. We performed a redundancy analysis to test the influence of dietary, medical, and habitual (smoking) factors upon the cyanide concentrations in the urine of studied adults, but the influences were mild at most (Figure 4). A non-parametric significance test in fathers and mothers as separate groups yielded significant differences only for cabbage in the group of mothers and broccoli among the fathers group (as measured with U Mann–Whitney test).

K9 and M7 samples noted the highest cyanide concentrations in the urine of both the mother and the father (>19 µg L^−^^1^), and in M7 the child’s meconium also displayed the highest, extreme level of cyanide (>24 µg L^−^^1^). This may indicate that the environmental factor common for the entire family played a crucial role. However, only one of these families harbored a heavy smoker (mother smoking six to ten cigarettes a day before pregnancy). Perhaps another factor that is not included in this study may be responsible for the levels of cyanide in parents’ urine and children’s meconium, higher than or similar to those caused by smoking. A possible influence could be smoke emission from the heating system in the house or exposure to polluted air (urban smog). In the above work, no symptoms were investigated or discussed in cases of continuous cyanide exposure. However, HCN concentrations in the range of 8–10 ppm in the breathing zone can cause headaches, chest pains, dizziness, fatigue, weakness, mental confusion, and, with prolonged exposure, even more severe symptoms [28].

## 4. Conclusions

The IC-PAD and PLE methods developed were successfully used to reliably determine the presence of cyanide ions in various human biological samples. The results of the analysis confirmed the presence of cyanide ions in the meconium samples, indicating the broad applicability of IC-PAD. In previous studies as well as in this study, urine samples, which do not require invasion into the body and are easy to obtain, were used [13,29,30]. Meconium samples have previously been used to analyze metabolites of alcohol, tobacco smoke, and other environmental xenobiotics, but not cyanide ions. Furthermore, the IC-PAD analysis for the detection of cyanide ions in meconium samples was proposed for the first time. The proposed method based on PLE was proven to be simple and more efficient. Considering the high reliability and the high degree of automation of the analytical process, the developed method described herein is applicable for routine use such as clinical control.

## Figures and Tables

**Table 1:** Survey data.

*n* = 22			
		**Mothers**	**Fathers**
**Age**	21–30	50%	18%
	31–40	45%	68%
	41<	5%	14%
**Education**	secondary education	9%	32%
	higher education	91%	68%
**Smoking status before pregnancy**	active	23%	27%
	non-smoking	77%	73%
**Smoking status during pregnancy**	active	5%	5% (in the presence of pregnant women)
	passive (at home)	14%	5%
	non-smoking	95%	95%
**Diet rich in almonds**	yes	68%	36%
***n* = 18**		**Newborn babies**	
**Gender**	Male	8	
	Female	10	
**Mass [cm]/Weight [g]**	Min	49	2550
	Max	67	3910
	Average	54.4	3333.89
**Method of delivery**	C-section	50%	
	Normal birth	50%	
**Method of feeding**	Breast milk	39%	
	Mixed	61%	
**Breast milk volume**	Large	28%	
	Sufficient	61%	
	Small	11%	

**Table 2:** Validation parameters of the IC-PAD method for the determination of the cyanide ion.

Sample	Linear Range [µg L^−1^]	Curve Equation	R	SD	CV [%]	Recovery [%]	LOD [µg L^−1^]
Urine	1–100	y = 0.169 x − 0.304	0.992	0.003	1.63	80	1.8
Meconium	1–50	y = 0.200 x − 0.165	0.986	0.033	0.37	79–83	0.58

**Table 3:** Concentrations of cyanide in the studied biological samples.

Type of Sample		Mean conc. [µg L^−1^]	SD [µg L^−1^]	Min conc. [µg L^−1^]	Max conc. [µg L^−1^]
Mother’s urine (*n* = 11)	Active smoker (*n* = 4)	14.3	9.19	8.42	25.2
	Passive smoker (*n* = 3)	6.57	2.82	1.99	8.69
	Control group (*n* = 4)	3.12	1.37	<LOD	4.85
Father’s urine (*n* = 13)	Active smoker (*n* = 5)	20.0	1.37	18.7	21.9
	Passive smoker (*n* = 3)	5.71	1.66	4.11	8.00
	Control group (*n* = 5)	1.71	0.571	<LOD	2.61
Meconium (*n* = 11)	Smoking or passive smoking parents (*n* = 4)	12.6	6.41	6.78	24.6
	Control group (*n* = 7)	2.74	0.985	<LOD	4.09

## Data Availability

The data presented in this study are available on request from the corresponding author. The data are not publicly available due to the authors’ ownership of the results.

## Sample Availability

Samples of the compounds are not available from the authors.
